# Changes in Hospital Care of Newborn Infants with Trisomy 13

**DOI:** 10.1007/s10995-025-04072-z

**Published:** 2025-02-22

**Authors:** Allison O. Ignatz-Hoover, Mohsen A. A. Farghaly, Anna Crist, Alshimaa Abdalla, Hany Aly, Mohamed A. Mohamed

**Affiliations:** 1 Neonatal Intensive Care Unit, Cleveland Clinic Children’s Hospital, 9500 Euclid Avenue #M31, Cleveland, OH 44195 USA; 2 Pediatrics Department, Aswan University, Aswan, Egypt

**Keywords:** NICU ethics, Palliative, Trisomy 13, Patau syndrome, Genetic, Counseling, Chromosomal disorder, Neonate

## Abstract

**Objective:**

To examine the changes over recent years in neonatal survival to discharge, prevalence of adverse events, surgical procedures, tracheostomy and/or gastrostomy tube (G-tube) placement, and length of stay (LOS) in infants with Trisomy 13.

**Methods:**

We identified newborn infants with Trisomy 13 in the National Inpatient Sample in the years 2003–2018. We calculated prevalence of associated conditions. We examined procedures done, and common adverse events associated with each condition, survival rates, and LOS. We also calculated changes in trends over the years.

**Results:**

The study identified 5792 newborn infants with Trisomy 13. Mortality during neonatal period was 58%. There was no significant change in mortality trends over the years, (p < 0.001). Average LOS was 10 (+ 34) days which had significantly increased over recent years (p < 0.001) and it was highest in conditions of NEC followed by gastrointestinal anomalies and sepsis. Among survivors, 2% were discharged with tracheostomy and 9% with G-tubes. LOS was significantly increased in association with these procedures.

**Conclusion:**

There was a slight decrease in infants admitted to neonatal intensive care units with Trisomy 13 over recent years. In-hospital neonatal mortality was unchanged. However, there was a significant increase in LOS, which was increased with procedures such as tracheostomy and G-tube placement, which may reflect a trend toward increasing interventions without a corresponding improvement in mortality.

## Significance

What is already known on this subject?

What this study adds?

The outcomes of infants born with Trisomy 13 have changed over recent years as more centers perform surgical procedures.

There is a significant increase in length of inpatient stay with increasing procedures such as tracheostomy and G-tube placement, but without a corresponding improvement in mortality.

## Introduction

Trisomy 13 (T13), also known as Patau syndrome, is a rare genetic condition that occurs when a person has an extra copy of chromosome 13 in some or all of their cells, and it is associated with multiple characteristic anomalies, severe neurocognitive deficits, and high rates of infant mortality (Springett et al., 2015). Some studies showed an increase in survival of infants diagnosed with T13, which was substantially higher among those undergoing cardiac surgery; in a United States population-based study using 1997–2007 data from nine states, 5-year survival was 9.7% (Meyer et al., 2016). A large Canadian study reported similar findings, utilizing 1991–2012 data from multiple databases in the single-payer health care system. One-year survival was 19.8%; furthermore, ten-year survival was 12.9% (Nelson et al., 2016). In a study using 1982–2008 data from the multicenter registry of the Pediatric Cardiac Care Consortium, median survival (conditioned to hospital discharge) was 14.8 years in 29 patients with T13 (Peterson et al., 2017).

T13 has been considered a lethal condition, but over the past decade a number of studies have demonstrated improved long-term survival as more infants received medical and surgical interventions (Janvier et al., 2012). At the same time, as arguably both a cause and an effect of this practice shift, there has been a dramatic shift in how pediatric ethicists and the larger pediatric community view decision-making for T13. A few major articles have appeared in the pediatric ethics literature in the past several years (Kett, 2020; Pyle et al., 2018), including a piece by John Lantos (2016), who argues that T13 compromises a gray area, in which survival rates are low but not too low, and neurocognitive impairment is severe but not total, and thus parental values should drive decisions.

Still, though, most studies on long-term survival for T13 have utilized data prior to more recent practices of surgical repair for complex cardiac defects and have not consistently reported on trends in healthcare utilization and in-hospital costs for this population. We hypothesize that there is a practice change in the management of T13 that involving more proactive procedures/interventions in the neonatal period. This study, then, aims to examine the changes over recent years in neonatal survival to discharge, prevalence of adverse events, surgical procedures, tracheostomy and/or G-tube placement, and LOS in infants with T13.

## Methods

We identified newborn infants diagnosed with T13 in the National Inpatient Sample (NIS), and its Kids' Inpatient Database (KID) subversion. NIS is a de-identified, publicly available inpatient healthcare database produced by the Health Cost and Utilization Project (HCUP). It contains data of more than 7 million hospital stays each year. In this study, we used the NIS for the years 2003–2018. All types of neonatal admissions were included whether they were direct admissions the neonatal intensive care units (NICUs), admissions from the emergency room or transfers from other hospitals. To avoid duplicate inclusion, infants who were transferred out of the delivery hospital were excluded.

Infants with T13 were identified in the dataset using the respective International Classification of Diseases codes—9th version (ICD-9) and 10th version (ICD-10. Similarly, infants developed postnatal condition, or adverse effects were identified using respective ICD-9 and ICD-10 codes. This study involved publicly available de-identified data; therefore, it was exempted from review by the Institutional Review Board (IRB).

We calculated prevalence of commonly associated conditions such as congenital heart disease (CHD), diaphragmatic hernias, central nervous system (CNS), pulmonary or gastrointestinal (GI) anomalies, or abdominal wall defects (AWD). We examined procedures done, and common adverse events such as sepsis, necrotizing enterocolitis (NEC), pulmonary hemorrhage, or pulmonary hypertension, mortality associated with each condition, and length of stay (LOS) in the overall sample and among those who were discharged home or transferred to a chronic care facility. We also calculated changes in trends over the years. We used logistic regression analysis to examine the association of different concurrent congenital anomalies or postnatal adverse effects while controlling for confounding variables such as birth weight, gestational age, sex, race, the presence of other previously mentioned congenital anomalies and medical conditions. We did not consider time series analysis methods as data used lacks the disposition of these infants after discharge.

## Results

**Fig. 1:**
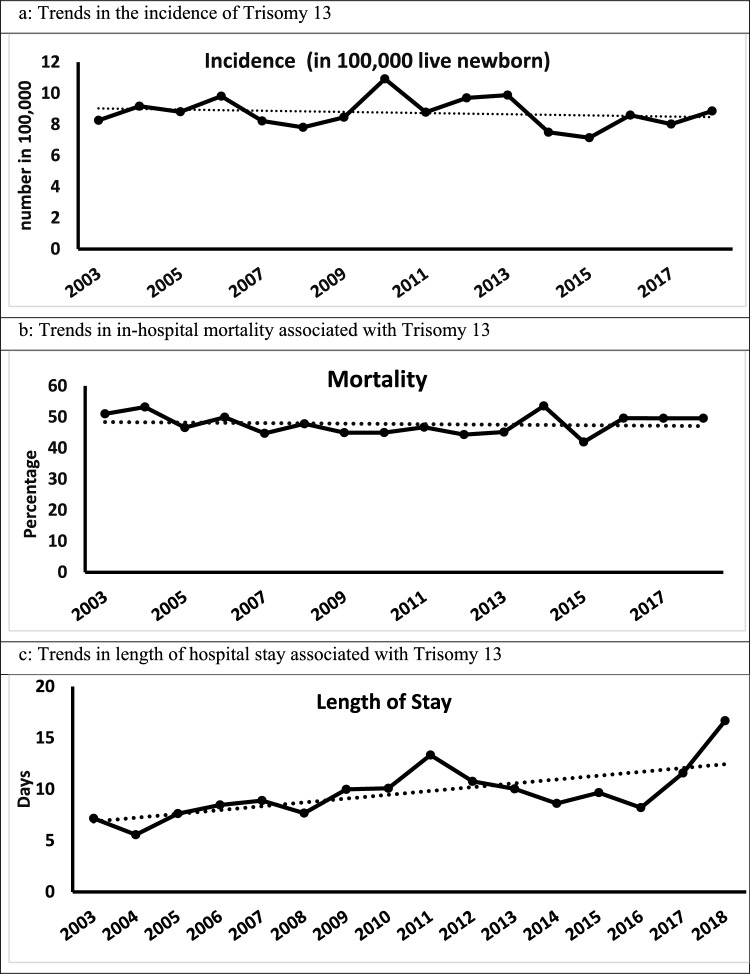
Trends in Trisomy 13 over 2003–2018 in the United States National Inpatient Database

The study identified 66,207,242 newborn infants. Of them, 5792 (approximately 0.01%) were diagnosed with T13 (8.7 in 100,000 live newborns). There was a mildly significant decrease in the prevalence of T13 over the years (p < 0.001, Fig. 1a). CHD were the most common associated anomaly (49%), followed by CNS anomalies (23%) and pulmonary hypertension (6.8%), Table 1.

**Table 1:** Characteristics of infants with Trisomy 13, associated mortality and length of stay in the United States National Inpatient Database in the years 2003–2018.^1^

	Frequency^1^ (95% CI)	Mortality^2^ (95% CI)	Length of Stay in days^3^	
			Overall	Among survivors^4^
Overall sample	n = 5792	58% (56–59)	10 (34)	16 (43)
Demographic characteristics				
Birth weight < 1500 g	11 (10–12)	87% (84–90)	8 (36)	39 (64)
Birth weight > 1500 g	89 (87–91)	56% (54–58)	10 (33)	15 (41)
Gestational age < 28 week	6 (5–7)	92% (85–99)	7 (47)	67 (151)
Gestational age > 28 week	94 (92–96)	54% (52–56)	10 (33)	15 (40)
Sex female	51 (50–52)	52% (51–53)	11 (37)	16 (47)
Sex male	49 (48–50)	64% (63–65)	90 (30)	15 (37)
Race/ethnicity				
Whites	35 (33–37)	56% (54–58)	8 (32)	14 (44)
African American	15 (12–18)	57% (55–59)	14 (46)	19 (55)
Hispanic Latino	20 (19–21)	58% (57–59)	9 (24)	14 (29)
Asian/Pacific Islander	2.4 (1.9–2.9)	66% (61–71)	8 (16)	12 (16)
Native American	0.6 (0.5–0.7)	48% (44–52)	15 (34)	21 (42)
Others	27 (25–29)	60% (59–61)	11 (33)	17 (43)
Associated congenital anomalies				
Congenital heart diseases	49 (47–51)	55% (54–56)	13 (35)	17 (39)
Nervous system anomalies	23 (21–25)	64% (61–67)	8 (26)	12 (33)
Hydrocephalus	5.0 (4–6)	69% (68–70)	10 (32)	15 (41)
Abdominal wall defects	4.5 (4–5)	65% (63–67)	4 (9)	3 (6)
Lung anomalies	4.0 (3.5)	77% (76–78)	12 (45)	32 (65)
Diaphragmatic hernia	2.0 (1–3)	75% (74–77)	90 (41)	20 (34)
Intestinal atresia	0.5 (0.3–0.6)	50% (48–52)	29 (54)	41 (61)
Common adverse outcomes				
Early or late sepsis	10 (9–11)	53% (52–54)	26 (58)	32 (70)
Pulmonary hypertension	6.8 (5.9–7.6)	59% (56–62)	21 (49)	27 (54)
Necrotizing enterocolitis	3.1 (2.5–3.7)	57% (56–58)	42 (77)	56 (87)
Pulmonary hemorrhage	0.4 (0.2–0.4)	91% (91–92)	28 (45)	n/a
Common procedures				
Gastrostomy tube	5.0 (4.6)	9.2% (8.9–9.5)	50 (82)	51 (80)
Tracheostomy tube	1.1 (0.8–1.5)	2.0% (1.5–2.5)	96 (94)	91 (98)

^1^All values are in percentages/proportions

^2^Mortality: In-hospital mortality till discharge including neonatal and post-neonatal mortality

^3^Length of hospital stay till death or discharge in days: mean + (sd)

^4^Survived only until discharge from the hospitals

Overall mortality, in-hospital, during the neonatal period was 58%. Table 2 Mortality was highest among those with pulmonary hemorrhage (91%), lung anomalies (77%), diaphragmatic hernia (75%), hydrocephalus (69%), abdominal wall defects (65%), other CNS anomalies (64%), and pulmonary hypertension (59%), Table 1. Furthermore, there was no significant change in mortality trends over the years, Fig. 1b and Table 3.

**Table 2:** Significant conditions associated with mortality in infants with Trisomy 13 in the United States National Inpatient Database in the years 2003–2018

	Odds ratios	95% confidence intervals	*p* value
Female sex	0.7	(0.5–0.8)	< 0.001
Gestational age < 28 week	5.6	(2.3–13.3)	< 0.001
Birth weight < 1500 g	4.2	(2.7–6.5)	< 0.001
Congenital heart diseases	0.8	(0.7–1.0)	0.05
Central nervous system anomalies	1.4	(1.0–1.8)	0.02
Lung anomalies	3.2	(1.4–7.2)	0.005

**Table 3:** Changes overtime in incidence, mortality and adverse events in Trisomy 13

	Overall sample	Infants born 2003–2006	Infants born 2015–2018	significance for trend over years^1^
Incidence of Trisomy 13 per 100,000 hospital admission	8.7	9.0	8.2	0.04
Congenital heart diseases	49%	43%	59%	< 0.01
Central nervous system anomalies	23%	16%	31%	< 0.01
Early or late sepsis	11%	10%	11%	0.12
Pulmonary hypertension	6.8%	7.0%	4.3%	< 0.01
Mortality^2^	58%	58%	58.%	0.57
Length of hospital stay^3^	10 (+ 34)	7 (+ 23)	12 (+ 50)	< 0.01

^1^Significance for trends is calculated over years of the study (2003–2018) in the United States National Inpatient Database

^2^Mortality: In-hospital mortality till discharge including neonatal and post-neonatal mortality

^3^Length of hospital stay till death or discharge in days: mean (sd)

Average LOS was 10 (+ 34) days. LOS had significantly increased over recent years (p < 0.001, Fig. 1c). Average LOS was highest in conditions of NEC followed by gastrointestinal anomalies (intestinal atresia and Hirschsprung disease), pulmonary hemorrhage and sepsis, Table 1.

We further compared the change in associated major anomalies during the first four years (2003–2006) and the last four years (2015–2018) of the study, that could potentially contribute to increased length of stay over time (Table 3). While mortality remained unchanged at 58%, LOS increased from 7 to 12 days (p < 0.01). The diagnoses of CHD and CNS anomalies increased in two time periods.

## Discussion

This study assessed the most recent survival data for the T13 populations, in addition to important measures of in-hospital healthcare utilization, using a large nationally representative hospital database. The study demonstrated that there was a slight decrease in infants admitted with T13 over recent years. That decline could be because of the increased prenatal genetic diagnosis and eventually elective termination of pregnancy and/or comfort care at birth.

In addition, the study demonstrated that the in-hospital neonatal mortality was unchanged in the US over almost two decades, 2003–2018. Meyer et al. (2016), found higher survival rate at 1 year of age, however, their sample size was much smaller, and they included infants with various genetic expressions, had lower prevalence of CHDs and GI anomalies, and selective termination of trisomy fetuses with major malformations or pregnancy complications.

Furthermore, this study showed that there was a significant increase in LOS, which was increased with procedures such as tracheostomy and G-tube placement, which may reflect a trend toward increasing interventions without a corresponding improvement in mortality. Our finding is consistent with a 2018 paper which did report that patients with T13 had higher inpatient medical costs, and longer LOS, but this utilized 1998–2009 data, prior to more recent trends in cardiac surgery and only captured data for a single state, Florida (Nguyen et al., 2018). The previous assumption that T13 is necessarily lethal in infancy created nonintervention practices that lead to extremely low survival, which might have furthered nonintervention practices, however, as more studies demonstrated potential survival > 1 year, more infants were offered life-sustaining medical treatment including surgical interventions (Nelson et al., 2012).^10^ Nevertheless, unfortunately the in-hospital mortality rate remained unchanged.

This study has several strengths. It demonstrated the changes in hospital care of neonates and infants with T13 using a recent national database in a cohort with a huge sample size representing the entire US with a robust number of sociodemographic and clinical covariates to control for potential confounders. Furthermore, the survival findings are representative as it is almost impossible to have errors in the NIS dataset related to survival and mortality (HCUP, 2024); and that might help parents and caregivers making decisions during antenatal consultations and/or counseling sessions. However, the study inherited a few limitations; this study could not provide a detailed long-term outcome after discharge from the hospital. In addition, it could not assess the association of survival with parent’s wishes. Yet, the study’s limitations have not impacted the results; as the huge sample size obtained through using the NIS datasets may overcome such limitations by creating a national average to the Trisomy 13 condition.

In conclusion, there was a slight decrease in infants admitted to neonatal intensive care units with T13 over recent years. In-hospital neonatal mortality was unchanged. However, there was a significant increase in LOS, which was increased with procedures such as tracheostomy and G-tube placement. This may reflect a trend toward increasing interventions without a corresponding improvement in mortality, which may be practical for geneticists and neonatologists for counseling families.

## Data Availability

The data that support the findings of this study are available from the National Inpatient Sample as part of the Healthcare Cost and Utilization Project. Restrictions apply to the availability of these data, which were used under license for this study. Data are available at https://www.hcup-us.ahrq.gov/.
